# Oxymetazoline and Blepharoptosis: A Case Series and Proposed Treatment Algorithm

**DOI:** 10.7759/cureus.88649

**Published:** 2025-07-24

**Authors:** Emanuella M Brito, Francisco Ferri, Titilopemi Odulanmi, Maria A Vera Silva, Juan P Cordero, Lindsey Foran, Martin I Newman, Andres G Sarraga

**Affiliations:** 1 Plastic and Reconstructive Surgery, Nova Southeastern University Dr. Kiran C. Patel College of Osteopathic Medicine, Fort Lauderdale, USA; 2 Plastic and Reconstructive Surgery, Cleveland Clinic Florida, Weston, USA; 3 Psychiatry, St. John’s Episcopal Hospital, Queens, USA; 4 Neuro-Ophthalmology, The University of Miami Bascom Palmer Eye Institute, Miami, USA; 5 Plastic Surgery, Florida International University, Miami, USA; 6 Plastic and Reconstructive Surgery, ARTIS Institute for Plastic Surgery, Aventura, USA

**Keywords:** alpha-1 agonist, blepharoptosis, eyelid elevation, oxymetazoline, plastic surgery, pstosis, visual field repair

## Abstract

Blepharoptosis, characterized by drooping upper eyelids, affects vision and daily life. Traditional management includes observation, pharmacological treatments, and surgery, though not all cases necessitate surgical intervention. Pharmacologic treatments such as apraclonidine and phenylephrine eyedrops have been used but are limited by significant adverse effects, including pupil dilation and ocular/systemic side effects. Recently, oxymetazoline ophthalmic solution, approved by the FDA in 2020, has emerged as a promising alternative, showing improvement in visual field and eyelid elevation with minimal adverse effects. This study aims to elucidate the utilization of oxymetazoline in patients with blepharoptosis by the senior author and to propose a treatment algorithm tailored for these patients. Oxymetazoline was administered to five patients with blepharoptosis in the absence of contraindications. Response to the medication was assessed clinically, and a treatment algorithm was devised. Following the oxymetazoline test, the subsequent course of action was determined as follows: a favorable response in the oxymetazoline test indicated a benefit from an internal ptosis repair with or without a blepharoplasty. A minimal or less than optimal response after the test suggested the necessity for external ptosis repair, possibly accompanied by blepharoplasty. Furthermore, in cases of asymmetric ptosis or unilateral ptosis, the test was employed on the affected or more ptotic eye initially to serve as an interdependence test. This also allowed the surgeon to determine if a unilateral or bilateral repair was necessary. Oxymetazoline offers a valuable therapeutic option for blepharoptosis, serving as both a diagnostic tool and a bridging therapy before surgery. Its extended duration of effectiveness and favorable safety profile make it an attractive choice for patients. The proposed treatment algorithm simplifies decision-making for clinicians and enhances patient satisfaction and quality of life.

## Introduction

Blepharoptosis is a condition where the upper eyelid droops below its normal position, potentially obstructing vision, impacting activities of daily living, and affecting facial aesthetics [1]. The severity of the blepharoptosis can be assessed with the margin-reflex-distance 1 (MRD1), which is the vertical distance from the upper eyelid margin to the corneal light reflex, with the average MRD1 being 4-5 mm [2]. This condition can occur unilaterally or bilaterally and is more common in adults, especially women over 40, with incidence rates ranging from 4.7% to 13.5% [3,4]. In children, it’s diagnosed in approximately 7.9 per 100,000 patients, often affecting the left side [5].

Upper eyelid elevation is controlled by the levator palpebrae superioris muscle (LP) and Müller’s muscle (MM). Due to its composition of skeletal and smooth muscle, the LP is under both voluntary and involuntary control. The LP muscle is innervated by the oculomotor nerve (CN III), which contains both motor nerves and preganglionic parasympathetic fibers. MM (also known as the superior tarsal muscle) assists the LP in maintaining eyelid elevation. MM receives innervation from the superior sympathetic cervical ganglion, placing it under involuntary control. MM is unique in that it is composed of smooth muscle fibers only (not skeletal) [1].

The causes of blepharoptosis may be congenital or acquired. Congenital causes may include: blepharophimosis syndrome; congenital third cranial nerve palsy; congenital Horner’s syndrome; or Marcus Gunn jaw-winking syndrome. Causes of acquired blepharoptosis include age-related involutional ptosis (also known as aponeurotic ptosis) or blepharoptosis secondary to myogenic, neurogenic, traumatic, or mechanical pathology. Of these, age-related involutional ptosis is the most common cause of acquired blepharoptosis, accounting for approximately 60% of referrals [2].

Identifying the specific cause of blepharoptosis with attention to the above is crucial for treatment. Traditional managements for age-related involutional ptosis include observation, pharmacological options like adrenergic eye drops, and surgery. For symptomatic individuals, surgical correction has been the primary approach for improving eyelid elevation and the upper visual field [6]. However, not all cases require surgery. Traditional pharmacologic approaches in management include the use of topical adrenergic eyedrops, such as apraclonidine and phenylephrine, which are a proven temporary measure to lift the upper eyelid by activating MM in most patients. Although beneficial, the adverse effects of phenylephrine causing significant pupil dilation and short duration limits its use. Additionally, apraclonidine presents with ocular side effects, such as decreased visual acuity, and systemic side effects such as contact dermatitis, making it also undesirable for patients [3]. However, topical oxymetazoline has recently emerged as a potential powerful pharmacological treatment for blepharoptosis.

Oxymetazoline is a non-selective alpha-adrenergic receptor agonist with rapid onset and prolonged effects [7]. It was approved by the FDA in July 2020 as an ophthalmic solution, showing significant improvement in the superior visual field and upper eyelid elevation in patients with acquired age-related involutional ptosis. Daily use is effective for up to eight hours with low adverse effects [3,4]. Besides its therapeutic benefits, oxymetazoline’s vasoconstrictor activity may also improve the appearance of the sclera by constricting hyperemic blood vessels.

We hold the belief that oxymetazoline represents an exceptional diagnostic and therapeutic option, one that is likely underutilized by ophthalmologists, oculoplastic and plastic surgeons, despite its evident advantages when contrasted with other pharmacological treatments available for blepharoptosis. In this manuscript, we outline the protocol for the utilization of oxymetazoline in patients with blepharoptosis by the senior author and, in doing so, offer a treatment algorithm tailored for these patients. This is an initial study based on five patients; a follow-up study with a larger sample size is planned.

## Case presentation

Treatment algorithm

**Figure 1:**
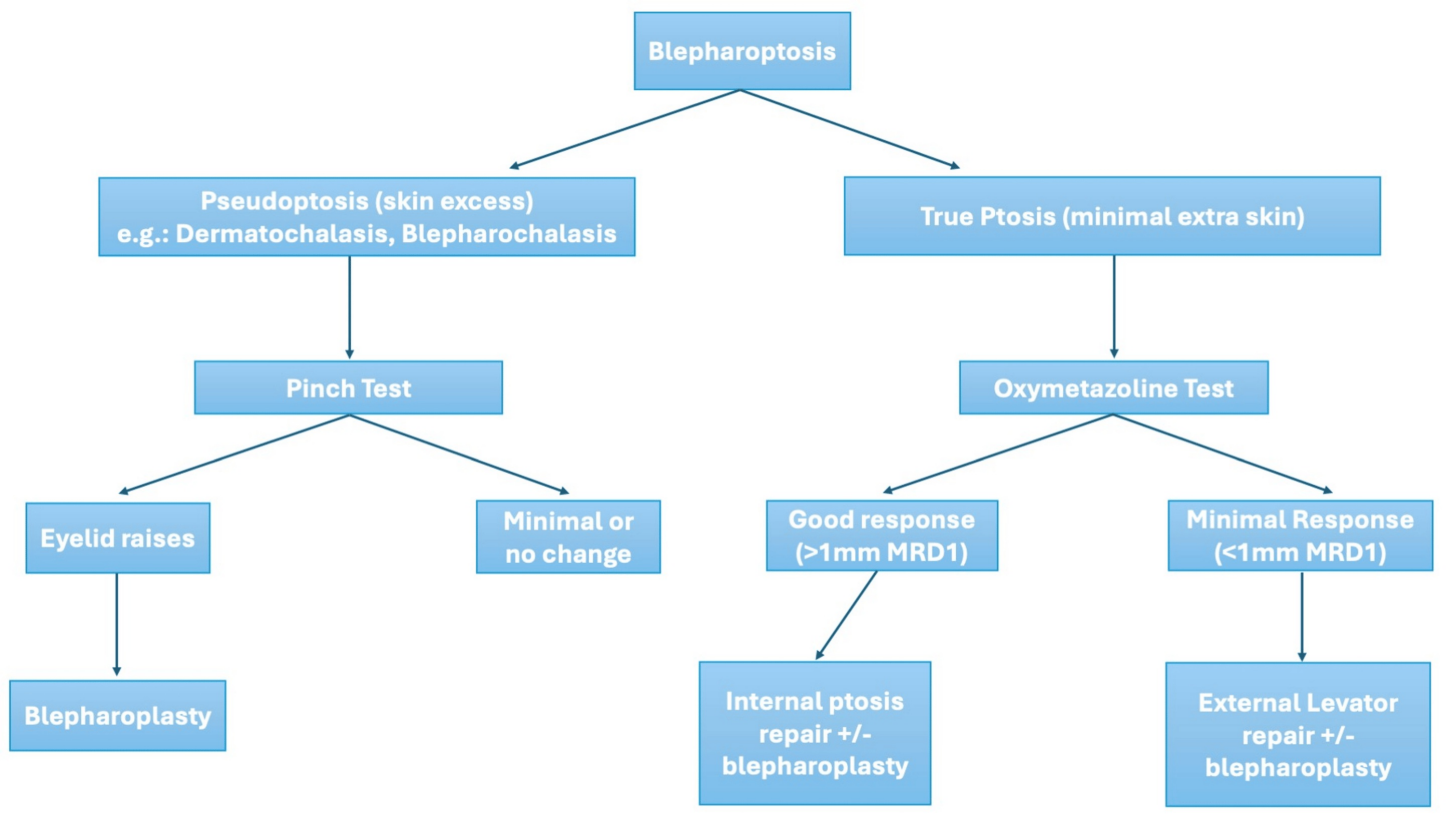
Proposed treatment algorithm for patients with blepharoptosis Image credits: authors EB, FF, and AS.

The use of oxymetazoline as a diagnostic and therapeutic tool in our practice is outlined below and illustrated in Figure 1. Oxymetazoline was offered to all adult patients presenting with blepharoptosis of various etiologies, such as congenital, traumatic, and most commonly in age-related involutional blepharoptosis. A discussion about its off-label use was held with all the patients. Contraindications included any patient who reported a contraindication based on the medication safety profile, as well as any patient taking alpha-adrenergic antagonists, beta blockers, or any other related medication to treat hypertension, heart diseases, or an enlarged prostate. Similarly, patients with Sjögren’s syndrome and glaucoma were considered not to be candidates due to the potential worsening of their underlying clinical condition. Finally, patients taking antidepressant medications (monoamine oxidase inhibitors) were counseled about how oxymetazoline may affect the absorption of the medication. 

Once a patient was considered a candidate, oxymetazoline hydrochloride ophthalmic solution 0.1% was employed as a preoperative investigation to assess the patient’s perception of potential improvement with surgery and as a diagnostic tool to evaluate for possible postoperative dry eye. As a preoperative investigation, we administered a single drop of oxymetazoline 0.1% ophthalmic solution to the affected eye, subsequently conducting a patient reevaluation at a 10-minute interval in a dark room while resting with eyes closed. A favorable response was considered to be an improvement of the MDR1 (i.e., lid recruitment) of at least 1 mm after 10 minutes. Based on the immediate response to the medication and the perceived outcome by the patient, we would proceed to assess and engage in a discussion with the patient regarding the potential need and options for surgical intervention. For a patient who experienced severe dry eye or exacerbation of the underlying condition, surgery was ruled out as an option.

All patients presenting with blepharoptosis due to the primary issue of excess upper eyelid skin (dermatochalasis), also referred to as pseudoptosis, underwent an initial assessment through a pinch test. If the eyelid exhibited elevation in response to the pinch test, the patient would qualify as a candidate for an upper lid blepharoplasty. In instances where the eyelid position remained unchanged after the pinch test, an oxymetazoline test was administered. Conversely, patients with a low lid level, minimal skin excess, and adequate levator excursion - indicative of true ptosis - were considered suitable candidates for the oxymetazoline test. Following the oxymetazoline test, the subsequent course of action was determined as follows: a favorable response in the oxymetazoline test indicated a benefit from an internal ptosis repair with or without blepharoplasty. A minimal or less than optimal response after the test suggested the necessity for external ptosis repair, possibly accompanied by blepharoplasty. Furthermore, in cases of asymmetric ptosis or unilateral ptosis, the test was employed on the affected or more ptotic eye initially to serve as an interdependence test. This also allowed the surgeon to determine if a unilateral or bilateral repair was necessary.

Once the surgical plan was established in collaboration with the patient, they were directed to continue using daily drops of oxymetazoline to the affected eye(s) as a bridging therapy prior to the scheduled surgery. For patients who were not deemed suitable candidates for surgical intervention or were still undecided about surgical correction, oxymetazoline treatment was administered to alleviate symptoms and enhance their quality of life.

In order to assess the success of this algorithm, five representative patients were evaluated and are presented below.

Patient 1

**Figure 2:**
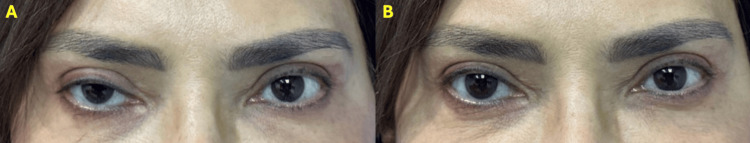
Patient 1 A) Female patient with right eye toxin-induced blepharoptosis OD. B) 10 minutes after oxymetazoline ophthalmic solution 0.1% in the right eye.

A 58-year-old female patient was seen in the office 10 days after injection of botulinum toxin in the forehead and glabellar area. The patient presented with toxin-induced blepharoptosis in the right eye. Oxymetazoline ophthalmic solution 0.1% was instilled into the right eye, showing significant improvement after 10 minutes (Figure 2).

Patient 2

**Figure 3:**
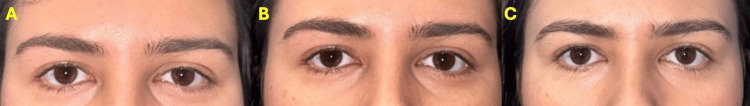
Patient 2 A) Female patient with OS mild congenital ptosis (left). B) Pretesting with oxymetazoline 0.1%. C) 6 weeks after OS internal ptosis repair.

A 23-year-old female patient presented with mild congenital ptosis in the left eye. Oxymetazoline ophthalmic solution 0.1% was used in the left eye as a diagnostic tool and yielded positive results after 10 minutes. The recommendation was made for her to undergo internal ptosis repair. The patient underwent the aforementioned surgery with satisfactory results from a 10 mm resection as seen during her 6-week follow-up (Figure 3).

Patient 3

**Figure 4:**
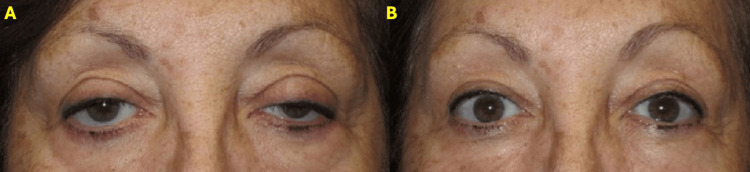
Patient 3 A) Female patient with OU severe ptosis. B) Patient pretesting with oxymetazoline 0.1% OU at 10 minutes.

**Figure 5:**
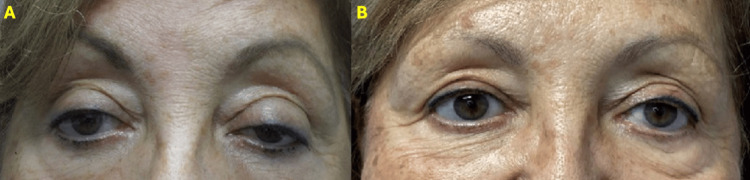
Patient 3 following internal ptosis repair A) Same patient as shown in Figure 4 before bilateral internal ptosis repair. B) Patient after 3 months post-op of bilateral internal ptosis repair with no skin excision.

A 71-year-old female patient with severe bilateral age-related involutional ptosis underwent the application of oxymetazoline ophthalmic solution 0.1% to both eyes with a favorable response after 10 minutes (Figure 4). Given these results, the recommendation was made for bilateral internal ptosis repair without blepharoplasty. 10 mm and 12 mm were resected from the right and left upper eyelids, respectively, with excellent results at the 3-month follow-up (Figure 5).

Patient 4

**Figure 6:**
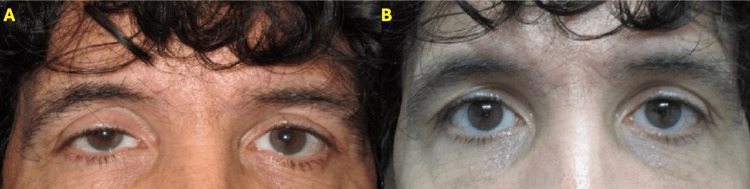
Patient 4 A) Male patient with OD posttraumatic ptosis. B) Patient after 10 min of oxymetazoline instilled into OD only.

A 56-year-old male patient presented with symptomatic posttraumatic ptosis in the right eye. Oxymetazoline ophthalmic solution 0.1% was applied to the right eye only, showing a good response. Based on the test results, this patient was an ideal candidate for unilateral ptosis repair (Figure 6).

Patient 5

**Figure 7:**
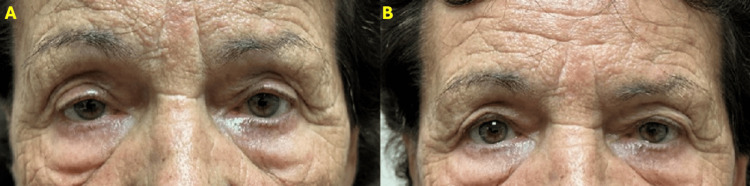
Patient 5 A) Female patient with symptomatic ptosis of her right eye. B) The patient, after 10 min of oxymetazoline in the right eye, with >1 mm MRD elevation. MRD: Marginal reflex distance

**Figure 8:**
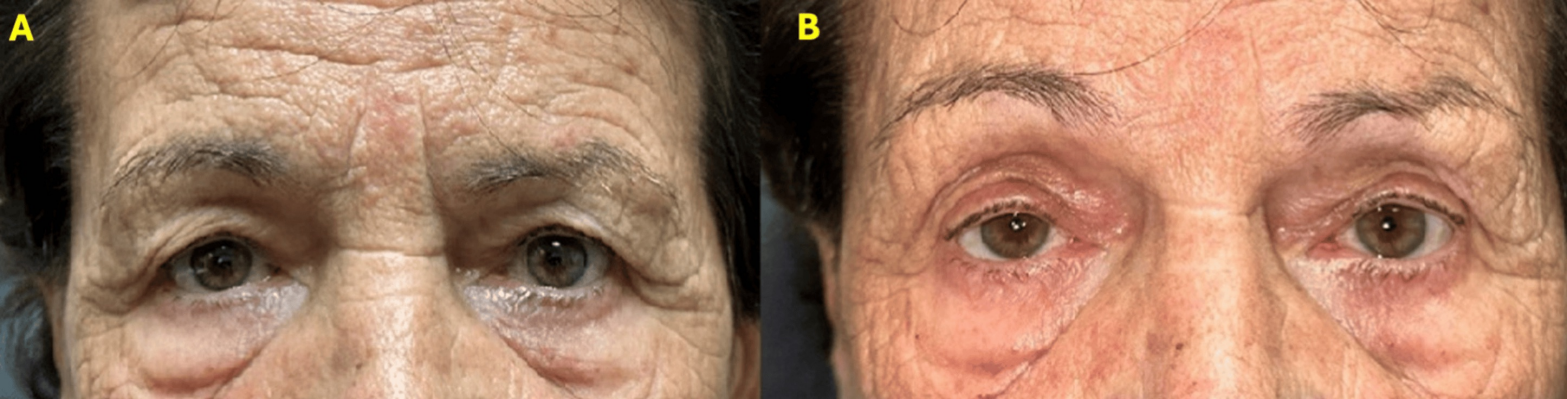
Patient 5 interdependence of the eyes A) Patient 5 with contralateral droop (reduction of MRD), confirming the interdependency of the eyes. B) The patient, after a drop of oxymetazoline was added to the left side. MRD: Marginal reflex distance

**Figure 9:**
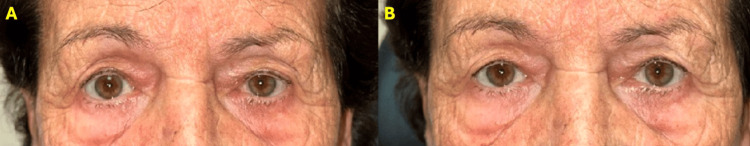
Patient 5 final results A) Patient 5 before surgery. B) Final results after bilateral internal ptosis repair.

A 78-year-old female patient presented with symptomatic age-related involutional ptosis of her right eye. Oxymetazoline ophthalmic solution 0.1% was instilled in her right eye with a satisfactory response after 10 minutes (Figure 7). However, she was noted to have a contralateral droop (reduction of MDR1), confirming the interdependency of the eyes. A drop was then added to the left eye with favorable results (Figure 8). The patient underwent bilateral internal ptosis repair with optimal results after an 8- and 10-mm upper eyelid resection on the left and right eyes, respectively (Figure 9).

## Discussion

In our practice, we have successfully employed oxymetazoline 0.1% ophthalmic solution in diagnostic and therapeutic applications. In a preoperative context, it serves as a diagnostic tool to ascertain whether patients perceive the desired improvements that would justify pursuing surgical correction, and for those in whom surgical intervention is contraindicated or not preferred by the patient.

Our proposed treatment algorithm is simple and effective, helping us to select appropriate candidates for surgery and providing them with the surgical option that best fits their needs. Furthermore, we have observed the medication’s benefits for patients with eyelid asymmetry, instances of toxin-induced blepharoptosis, and in scenarios necessitating a combination of treatment modalities. Another added benefit was that the use of the oxymetazoline drops would unmask the dry eye patient who would not be able to tolerate a ptosis repair and potentially spare the patient from a permanent dry eye situation. In summary, we have determined that oxymetazoline proves to be an effective diagnostic and therapeutic option for blepharoptosis of various etiologies. Our patients have reported elevated levels of satisfaction and a notable improvement in their quality of life. The commendable safety profile of oxymetazoline renders it a highly advantageous option for the pharmacological treatment of blepharoptosis, particularly when compared to alternatives such as phenylephrine and apraclonidine.

Our findings also align with the existing body of literature. A recently published double-blinded randomized controlled trial conducted at the University of Miami Bascom Palmer Eye Institute, involving 114 patients afflicted with severe blepharoptosis, revealed a significant increase in the MRD1, palpebral fissure height, decreased ocular redness, and enhanced perceived eye aesthetics after the utilization of topical oxymetazoline 0.1% compared to a placebo group [8]. It is noteworthy that 12.2% of the subjects administered oxymetazoline experienced adverse events, most frequently reporting eye dryness and headaches, although a similar proportion of participants in the control group also reported experiencing headaches [8]. These findings are consistent with prior publications, demonstrating a substantial improvement in visual field deficits and an increase in MRD1 following the administration of oxymetazoline ophthalmic solution compared to placebo [9,10].

Likewise, Bacharach and colleagues conducted an analysis involving the pooling of data from two prospective, randomized, placebo-controlled studies analyzing the change in MRD1 after instillation of oxymetazoline 0.1% ophthalmic agent for the treatment of acquired blepharoptosis in adult patients [11]. The investigation revealed a significant increase in MRD1 at the 5- and 15-minute intervals following instillation on days 1, 14, and 42 of the treatment, thus substantiating that oxymetazoline confers both rapid and sustained elevation of the upper eyelid [11].

The safety profile of daily topical administration of oxymetazoline in patients with blepharoptosis was analyzed by Wirta et al. [12]. A pooled analysis of four randomized clinical trials indicated that the incidence of treatment-emergent adverse events was similar among participants using oxymetazoline compared to the placebo group. Nearly all adverse events were mild to moderate, and most were not suspected of being treatment-related. The authors concluded that the medication is safe and effective when used for 14-84 days [12].

Iatrogenic blepharoptosis resulting from periocular botulinum toxin treatment is a condition that has shown an increasing incidence in recent years, posing challenges for both patients and practitioners. Bernardini and colleagues reported a case series in which patients with botulinum toxin-induced ptosis were effectively managed using topical oxymetazoline [13]. In our private practice, even though a rare occurrence, we have successfully treated two patients with this issue.

In addition to its application in treating acquired blepharoptosis, topical oxymetazoline has been suggested to potentially exhibit a synergistic effect with botulinum toxin when employed in combination for the reduction of blepharospasm [14]. Furthermore, it has demonstrated successful application in animal models for the treatment of non-infectious conjunctivitis, a prevalent etiology of acute red eyes [15].

Our study presents certain limitations. Firstly, it describes the experience of a single surgeon within a single medical facility. Secondly, it lacks objective data pertaining to our results. The principal objective of this paper was to elucidate the utilization of oxymetazoline in the daily practice of the senior author, an oculoplastic-trained plastic surgeon, and to propose a treatment algorithm for patients with blepharoptosis. Future investigations will be conducted incorporating objective data acquired from our patient cohort. Thirdly, it is important to acknowledge that some of the publications utilized in the literature review had sponsorship from industry sources. Future studies should also include a systematic review and possible meta-analysis comparing oxymetazoline to the current pharmacological standard of care for the treatment of blepharoptosis. Nonetheless, despite these constraints, our findings remain in accordance with the current body of literature.

## Conclusions

The utilization of oxymetazoline ophthalmic solution 0.1% serves as an educational resource for patients in preparation for surgery, as well as a sustainable treatment for ptosis patients who are either unsuitable candidates for surgery or have a preference against surgical intervention. Furthermore, the application of this medication can be extended to include asymmetric patients, individuals experiencing toxin-induced blepharoptosis, and cases involving combination treatments. The medication’s extended duration of effectiveness and favorable safety profile underscore its value as a valuable addition to the aesthetic and functional surgery armamentarium. The notably low incidence of adverse events associated with this medication renders it an exceptionally attractive choice.
